# Stroke or No Stroke: A Case Report of Bilingual Aphasia

**DOI:** 10.5811/cpcem.2021.4.51206

**Published:** 2021-06-21

**Authors:** Matthew Gray, Jacob Ernst, Simeon Ashworth, Ronak Patel, Kyle Couperus

**Affiliations:** Madigan Army Medical Center, Department of Emergency Medicine, Joint Base Lewis-McChord, Washington

**Keywords:** Case report, bilingual aphasia, stroke

## Abstract

**Introduction:**

Bilingual aphasia is an atypical stroke presentation in the multilingual patient where an isolated aphasia occurs in one language while the other remains unaffected.

**Case Report:**

A multilingual male presented to the emergency department with expressive aphasia to English but who was still able to speak fluently in French. Receptive English was preserved. While his National Institute of Health Stroke Scale score was technically zero, his pure aphasia component qualified him as an exception. He regained some repetitive English, so fibrinolyitic therapy was not initiated.

**Conclusion:**

Bilingual aphasia is an indication for fibrinolysis given the impact that a pure aphasic stroke has on quality of life.

## INTRODUCTION

Language processing is one of the most unique, vital, and complex functions of the human brain. This involves the planning of speech, the motor act of its articulation, the auditory reception of the response, as well as the comprehension of the meaning behind vibrations in the air. The brain’s ability to understand the world by giving image to thought and feeling through rhetoric is dependent upon language. In the same way it serves as the mortar that binds social groups together through its sharing. Disturbances in language can be devastating as they infantilize the brain, returning it to a time when it did not comprehend the world around it.^1^

## CASE REPORT

A 93-year-old male presented to the emergency department aphasic to English but still fluent in French. Two hours prior, the Creole-born Louisiana native felt suddenly lightheaded without more definite subjective symptoms. The multilingual patient understood English, confirmed through a French translator; however, when asked to repeat English words like “house” his response was the correct French word “*maison*.” He had no motor deficits. Per the translator, he was neither dysarthric nor disarticulate in his speech but was speaking with an “English” accent, the colloquial term used by French Canadians for their English-speaking countrymen. Most noteworthy, the patient was not speaking his native Creole but rather true French, a learned language, but one in which he was fluent. His friend noted a similar episode had occurred several months before and lasted about an hour, but they had not sought treatment as it resolved.

With a National Institute of Health Stroke Scale (NIHSS) of zero, and his three-hour fibrinolytic window rapidly closing, the most pressing question was whether to administer fibrinolytic therapy for an isolated speech deficit. Vital signs were only notable for moderate hypertension, 158/72 millimeters of mercury; the remainder of his neurologic testing was normal with symmetric and appropriate reflexes, strength, sensation, and cerebellar testing. The patient underwent rapid computed tomography without any evidence of hemorrhage. His glucose and electrolytes were normal, and his electrocardiogram was unremarkable. He bore no evidence of acute trauma, and he had no identifiable infection. Over the hour of his workup, the patient’s expressive English began to return, providing a relative contraindication to fibrinolytic therapy, as his clinical course was more suggestive of a transient ischemic attack. He retained his memory of the entire event, unsure of why it had occurred. Follow-up magnetic resonance imaging did not demonstrate an acute stroke, and the patient was discharged by the neurology service the following day on optimal medical therapy.

## DISCUSSION

Similar presentations of bilingual aphasia have been found to be the result of stroke, focal seizure, migraine, concussion, and illness.^2^ Notably, patients undergoing a stroke have been found to revert to speaking only their learned language in a phenomenon known as bilingual aphasia, while some are left only with their native language.^3^ Theories exist as to why this occurs especially given the discrepancy among the various cases; however, no definitive explanation exists. Likely, hypoperfusion of Broca’s area results in selective processing and expression along the motor arm of speech.^4^,^5^ With Wernicke’s area unaffected, comprehension of speech, regardless of the language, is unaffected. Total loss of a language would suggest a different etiology such as hypoperfusion of the whole dominant hemisphere or even in the whole brain.^6^

Alternative case series describe a slightly different phenomenon known as foreign accent syndrome in which a patient suffers a stroke and thereafter speaks with what bystanders deem to be a foreign accent.^7^ This is actually a form of mild dysarthria giving only the appearance of accented speech rather than true transition to a new phonation. In this case, however, it was clarified with the Québec interpreter that by “English” he meant the colloquial term French Canadians use for their English-speaking countrymen, rather than a “British” accent. The patient spoke as expected for someone who had learned traditional French as a second rather than primary language.

Stroke care is highly protocolized with screening questions to assess the indications and contraindications of fibrinolytic therapy.. Fibrinolysis is generally contraindicated in mild strokes classified by an NIHSS of less than six.^8^ The rationale is that the benefit of fibrinolysis is typically decreased with very small strokes, just as the risk is significantly increased in very large strokes with an NIHSS greater than 25.^9^ Aphasia is considered an exception to these limitations given the degree of impact it has on the patient’s quality of life.^10^,^11^ Not only is language vital to quality of life but isolated aphasia can portend more serious unmanifested ischemia.^10^ Thus, fibrinolysis is recommended in patients with isolated aphasia, especially in a language of daily use.^11^,^12^

## CONCLUSION

Although the patient in this case did not ultimately require fibrinolytic therapy, this presentation highlights an important exception to the common inclusion/exclusion criteria for fibrinolytic therapy in stroke. Although rare in their presentation, pure aphasic strokes can affect all or some of a patient’s ability to comprehend or express language. Thus, in an appropriate candidate, fibrinolytic therapy is recommended by the literature for these situations.

**CPC-EM Capsule**

What do we already know about this clinical entity?

*Bilingual aphasia is a unique stroke presentation in which a singular language, rather than all language function, is lost*.

What makes this presentation of disease reportable?

*Despite not meeting typical stroke scale criteria for thrombolysis, bilingual aphasia is considered a thrombolytic candidate*.

What is the major learning point?

*Bilingual aphasia should be evaluated and worked up as a stroke as this is a time-sensitive diagnosis for intervention*.

How might this improve emergency medicine practice?

*Rapid diagnosis of bilingual aphasia can lead to quicker activation of code stroke pathways and improve some patients’ long-term outcomes*.

## References

[1] Perani D, Farsad M, Ballarini T (2017). The impact of bilingualism on brain reserve and metabolic connectivity in Alzheimer’s dementia. Proc Natl Acad Sci U S A.

[2] Khachatryan E, Vanhoof G, Beyens H (2016). Language processing in bilingual aphasia: a new insight into the problem. Wiley Interdiscip Rev Cogn Sci.

[3] Lorenzen B, Murray LL (2008). Bilingual aphasia: a theoretical and clinical review. Am J Speech Lang Pathol.

[4] Kuzmina E, Goral M, Norvik M (2019). What influences language impairment in bilingual aphasia? A meta-analytic review. Front Psychol.

[5] Morelli N, Rota E, Colombi D (2019). Functional MRI provides insights into language organization of bilingual aphasia. Neurology.

[6] Miller DJ, Simpson JR, Silver B (2011). Safety of thrombolysis in acute ischemic stroke: a review of complications, risk factors, and newer technologies. Neurohospitalist.

[7] Barreto SDS, Ortiz KZ (2020). Speech in the foreign accent syndrome: differential diagnosis between organic and functional cases. Dement Neuropsychol.

[8] Brown MD, Burton JH, Nazarian DJ (2015). Clinical Policy: Use of intravenous tissue plasminogen activator for the management of acute ischemic stroke in the emergency department. Ann Emerg Med.

[9] Chapman SN, Mehndiratta P, Johansen MC (2014). Current perspectives on the use of intravenous recombinant tissue plasminogen activator (tPA) for treatment of acute ischemic stroke. Vasc Health Risk Manag.

[10] Nesi M, Lucente G, Nencini P (2014). Aphasia predicts unfavorable outcome in mild ischemic stroke patients and prompts thrombolytic treatment. J Stroke Cerebrovasc Dis.

[11] Denier C, Chassin O, Vandendries C (2016). Thrombolysis in stroke patients with isolated aphasia. Cerebrovasc Dis.

[12] Menichelli A, Furlanis G, Sartori A (2019). Thrombolysis’ benefits on early post-stroke language recovery in aphasia patients. J Clin Neurosci.

